# Attenuated serum vasoactive intestinal peptide concentrations are correlated with disease severity of non-traumatic osteonecrosis of femoral head

**DOI:** 10.1186/s13018-021-02486-3

**Published:** 2021-05-20

**Authors:** Ming Liu, Gan Zhao, Biao-Fang Wei

**Affiliations:** 1 Guangzhou University of Chinese Medicine, Guangzhou, Guangdong Province China; 2 Department of Pain, Linyi People’s Hospital, Linyi, Shandong Province China; 3 Department of Sports Medicine, Linyi Traditional Chinese Medicine Hospital, Linyi, Shandong Province China; 4 Department of Orthopedics, Linyi People’s Hospital, Jie Fang Road East, No.27, Linyi, 276003 Shandong Province China

**Keywords:** Non-traumatic osteonecrosis of the femoral head, Disease severity, Vasoactive intestinal peptide

## Abstract

**Background and objective:**

The neuropeptide vasoactive intestinal peptide is a 28-amino acid neuropeptide that has been shown to stimulate bone repair and angiogenesis. The purpose of this study was to explore the potential role of serum VIP concentration in osteonecrosis of femoral trauma (ONFH).

**Methods:**

One hundred five patients diagnosed with non-traumatic ONFH and 103 healthy individuals were enrolled in our study. Serum VIP, tumor necrosis factor-α (TNF-α), interluekin-1 beta (IL-1β), and macrophage colony-stimulating factor (M-CSF) levels also were detected using the commercial ELISA kit. Radiographic progression was evaluated using FICAT classification. The clinical severity of ONFH was assessed by visual analog score (VAS) and Harris Hip Score (HHS). Receiver-operating characteristic (ROC) curve was performed to test the potential diagnostic value of VIP in radiographic progression.

**Results:**

The serum VIP level of patients with non-traumatic ONFH was significantly lower than that of healthy controls. There was no significant difference between the alcohol group, the steroid-induction group, and the idiopathic group. Serum VIP levels were significantly higher in ONFH patients with femoral head pre-collapse stage than collapse stage. Serum VIP levels were significantly lower. FICAT 4 non-traumatic ONFH patients had significantly lower serum concentrations of VIP when compared with FICAT 3 and FICAT 2. Moreover, serum VIP levels were significantly lower in ONFH patients with FICAT 3 than FICAT 2. Serum VIP levels were negatively related to FICAT stage. In addition, serum VIP levels were negatively associated with VAS score and positively associated with HHS score. Last, we found serum VIP levels were negatively associated with serum TNF-α and IL-1β levels. ROC curve analysis indicated that decreased serum VIP could serve as a decent biomarker with regard to the diagnosis of radiographic progression.

**Conclusion:**

Attenuated serum VIP concentrations are correlated with disease severity of non-traumatic ONFH. Decreased serum VIP may serve as a potential indicator of non-traumatic ONFH.

## Introduction

Non-traumatic femoral head necrosis (ONFH, also known as avascular necrosis of the femoral head and aseptic femoral head necrosis) is a common but difficult-to-recover osteonecrosis disease, caused by abnormal blood supply to bone tissue, leading to bone tissue death, structural reconstruction, and collapse [1]. Recent epidemiological surveys have indicated that ONFH may afflict > 20 million of people worldwide, 5–7.5 million in China [2]. According to statistics, about 80% of patients will cause the femoral head to completely collapse, which is a huge challenge for orthopedic surgeons [3]. Among non-traumatic ONFH patients, high-dose corticosteroid therapy for inflammatory diseases and alcohol-abuse were reported to be the two main risk factors for non-traumatic ONFH [4]. These factors may lead to some potential pathogenesis of ONFH, including intraosseous hypertension, fat metabolism disorder, intravascular coagulation, microvascular endothelial cell damage, and osteoblast and bone cell apoptosis [5], which may cause final necrosis of femoral head.

In general, non-traumatic ONFH is usually asymptomatic in the early stages and difficult to diagnose. In many cases, the femoral head was collapsed when patients have symptoms [6]. Once the femoral head collapses, ONFH is difficult to reverse [7]. Although improvements in total hip replacement (THA) and hip joint protection technology have improved the quality of life of patients with non-traumatic ONFH, many such patients may face the psychological and economic burden of revision and replacement surgery after THA. In addition, more and more clinical evidence shows that joint-sparing surgery can bring successful results in the pre-collapse stage of ONFH [8]. Therefore, in order to effectively prevent collapse and delay disease progression, it is very important to find new biomarkers to diagnose non-invasive ONFH.

Vasoactive intestinal peptide (VIP) is a 28-amino acid neuropeptide that belongs to a glucagon/secretin superfamily. VIP has three types of receptors including receptor type 1 (VPAC1), VIP receptor type 2 (VPAC2), and the pituitary adenylate cyclase activating polypeptide (PACAP) type 1 (PAC1) receptor [9, 10]. VIP is expressed in the lung, small intestine, central nervous system neurons, and other tissues, and plays a key role in stimulating heart contraction and vasodilation, promoting neuroendocrine-immune communication, increasing glycogen decomposition, lowering arterial blood pressure, and other biological functions [10]. VIP has anti-inflammatory and immunomodulatory effects. Regulating its level is considered to be a potential drug candidate for the treatment of inflammation and autoimmune diseases, such as pancreatitis, septic shock, inflammatory bowel disease, lipopolysaccharide (LPS)-induced acute inflammation, and arthritis [10]. VIP can also regulate nuclear factor kappa B ligand receptor activator (NF-κB); osteoprotegerin (OPN) and macrophage colony-stimulating factor (M-CSF) promote the differentiation of osteoblasts and reduce the occurrence of osteoclasts [11, 12]. Shi et al. found in another study that VIP can promote the osteogenic differentiation of bone marrow mesenchymal stem cells and promote the repair of rat skull defects [13].

All these studies above indicated that VIP was a benefit for bone formation and repair. However, there were no works available before investigating the potential role of VIP in non-traumatic ONFH. Therefore, based on the findings above, this study was aimed to explore the potential serum VIP levels with the disease severity of non-traumatic ONFH.

## Study subjects and methods

### Study subjects

From March 2020 to April 2021, a total of 105 hospitalized non-traumatic ONFH patients in Linyi people’s hospital were recruited in the study. The diagnosis of ONFH was through the history and physical examination, X-ray, and MRI scans of the hips and based on the following criteria by Sugano [14]: (1) The symptoms and signs of clinical non-traumatic ONFH include hip joint pain, limited internal rotation, and sometimes knee and thigh pain. (2) The low-intensity band appears in the T1 image, and the “double-line sign” appears in the T2-weighted image. (3) CT scans and radiographs of the pelvis showed hardening of the edges and “crescent” femoral heads. (4) The radionuclide bone scan showed “cold-in-hot”. (5) Histological examination showed evidence of trabecula and bone marrow necrosis. The diagnosis of non-traumatic ONFH is when the patient exhibits two or more of these five criteria. In this study, patients with steroid-induced ONFH have a history of long-term daily intake of steroids exceeding 16 mg, or continuous high-dose steroid pulse treatment for more than 1 week before the onset of these symptoms. Alcohol-induced ONFH patients have a clinical history of high persistent alcohol intake (more than 5 years, the average actual alcohol intake is 100 g per day). Patients with traumatic goiter, sickle cell disease, end-stage renal disease, current GC treatment, and organ transplantation are excluded. In addition, all enrolled patients with osteoarthritis in one or both the hip or knee joints were also excluded. At the same time, 103 healthy control groups with age, gender matching, and regular physical examination were selected as controls. All patients and healthy volunteers gave written informed consent. The current study was approved by the Ethics Committee of Linyi People’s Hospital.

### Post hoc statistical power calculation

Use the Power and Sample Size Calculators (http://powerandsamplesize.com) to calculate statistical power (1-β) to obtain data on different average VIP levels, standard errors, and the number of enrolled patients in each group [15]. When > 0.8, the statistical power is considered strong. Calculated as follows:

$$ 1-\beta =\varphi \left(z-{{z_{1-}}_{\alpha}}_{/2}\right)+\varphi \left(-z-{{z_{1-}}_{\alpha}}_{/2}\right),z=\left({\mu}_{\mathrm{A}}-{\mu}_{\mathrm{B}}\right)/\sigma \left(\frac{1}{n_A}+\frac{1}{n_B}\right) $$

### Laboratory examination

Blood samples from all patients and controls were collected after overnight fast in plain tubes containing a separation gel. The blood samples were processed to collect serum and stored at −80°C until examination. Double-blinded quantitative measurement of VIP in serum was done by commercially available ELISA kit (Cusabio, Wu Han, China, Cat No. CSB-E08354h) based on the manufacturers’ instructions. The detection range was from 15.6 to 1000 pg/mL. According to the manufacturer, the intra-assay CV was < 8%, and the inter-assay CV was <10%. Serum TNF-α (Abcam, Cambridge, UK), IL-1β (Abcam), and M-CSF (Abcam) levels were also examined. All measurements were taken three times for each sample, and the results were averaged.

### Definition of radiographic progression

The radiographic severity was defined by FICAT classification [16]: stage 0, inconspicuous/normal findings; stage 1, inconspicuous findings or minor changes (slight patchy osteoporosis, blurring of trabecular pattern, subtle loss of clarity); stage 2, diffuse/focal radiological changes (osteoporosis, sclerosis, cysts), subchondral fracture (crescent sign), or segmental flattening of femoral head (out-of-round appearance); stage 3, broken contour of femoral head, bone sequestrum, joint space normal; stage 4: flattened contour of femoral head, decreased joint space collapse of femoral head, acetabular osteoarthritic changes. In our study, ONFH patients with FICAT phase ≥ 2 were enrolled. The results of radiographs were read and evaluated by two experienced radiologist who were blinded to the patients. The *Kappa* value was used to examine the consistency of the results, and *Kappa* value ≥ 0.8 was regarded significant.

### Determination of symptomatic severity

The evaluation of symptomatic severity of non-traumatic ONFH was performed by visual analog scale (VAS) and Harris Hip Score (HSS). VAS was the most common pain-assessing tool to evaluate pain. The patients were asked to draw marks on the horizontal line according to their own feelings to indicate the degree of pain from 0 to 10 points: 0 indicates no pain and 10 suggests extreme pain [17]. The Harris Hip Score (HHS) was used to assess global pain, function, deformity, and range of motion [18]. The pain item measures pain severity and its effect on activities and need for pain medication. The function item is divided into daily activities (stair use, using public transportation, sitting, and managing shoes and socks) and gait (limp, support needed, and walking distance). The deformity item observes hip flexion, adduction, internal rotation, and extremity length discrepancy while the range of motion (ROM) domain assesses hip ROM. The range of motion item consists of 6 motions that are graded based on the arc of motion possible. Each range of motion gradation is assigned an index factor and a maximum possible value, which are used to calculate arc of motion points [18]. The HHS was ranked from 0 (worst functional outcome and maximum pain) to 100 (best functional outcome and the least amount of pain) points. The interpretation of outcome using the modified HHS included the following: <70 (poor result), 70–79 (fair result), 80–89 (good result), and >90 (excellent result).

### Statistical analysis

The data were analyzed utilizing the GraphPad Prism 8.0 statistical software. All obtained data are shown as mean ± standard deviation or median. The normality of continuous variables was assessed through the Shapiro–Wilk test. Demographic data between non-traumatic ONFH patients and healthy individuals were compared using Chi-square tests and/or unpaired Student’s t-tests, where appropriate. Comparisons between FICAT subgroups were performed using one-way analysis of variance followed by Tukey or Tamhane post hoc test. Spearman or Pearson’s correlation coefficient was employed to determine correlations between VIP levels and disease severity. The area under curve (AUC) of all tested markers was determined through receiver operating characteristic (ROC) curve. Statistical significance was accepted for *P* <0.05.

## Results

### Demographic data

The demographic data are depicted in Table 1. The 105 patients included 70 males and 35 females with an average age of 48.8± 10.2. The 103 healthy subjects included 66 men and 37 women with an average age of 47.7 ± 9.4. No significant differences in age and sex distribution and BMI were observed between ONFH patients and healthy controls. After calculation, the statistical power was 0.86, suggesting that the sample size of 105 was sufficient to obtain the conclusion.

**Table 1:** Demographic data for ONFH patients and healthy controls

	ONFH patients (n=105)	Healthy controls (n=103)	***P*** value
Age (Y)	48.8±10.2	47.7±9.4	0.413
Sex (F/M)	35/70	37/66	0.155
BMI (kg/m^2^)	23.1±2.0	23.2±2.6	0.168
Disease duration (M)	85 (5–210)	/	
VAS scores	5.0±1.7	/	
HSS scores	68.8±12.1	/	
FICAT stage (2/3/4)	34/37/34	/	
Serum VIP levels (pg/mL)	209.6±37.2	268.5±41.0	<0.001
Serum TNF-α levels (pg/mL)	1.41±0.10	1.03±0.04	<0.001
Serum IL-1β levels (pg/mL)	3.45±0.12	1.05±0.05	<0.001
Serum C-MSF levels (pg/mL)	70.27±12.16	67.05±14.14	0.179

All data are given as mean value ± SD or median

### Serum VIP levels in ONFH patients

**Fig. 1:**
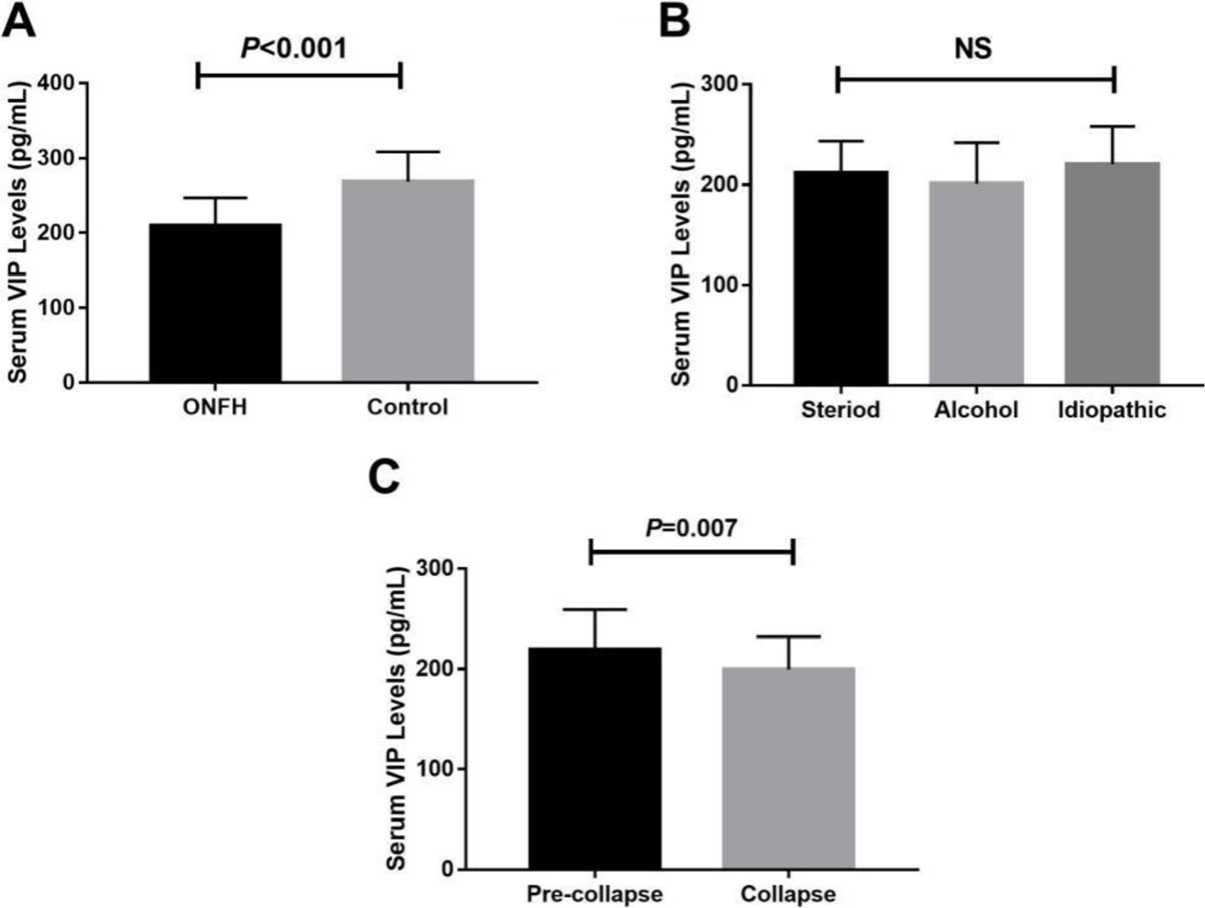
**A** Comparison of serum VIP levels between non-traumatic ONFH and healthy control. **B** Comparison of serum VIP levels among steroid-induced group, alcohol-induced group, and idiopathic group. **C** Comparison of serum VIP levels between femoral head pre-collapse ONFH patients and femoral collapse ONFH patients

Forty patients were alcohol-induced, 40 steroid-induced, and 25 idiopathic osteonecrosis. Serum VIP levels were significantly lower in non-traumatic ONFH patients compared with healthy controls (209.6±37.2 pg/mL vs 268.5±40.1 pg/mL, *P* <0.001) (Fig. 1A). There were no significant differences in serum VIP level among the ONFH patients with different etiologies (steroid-induced group 211.7±31.9 pg/mL vs alcohol-induced group 201.1±41.0 pg/mL vs idiopathic group 220.7±37.8 pg/mL, F=2.227, *P=*0.113) (Fig. 1B). In addition, serum VIP levels were significantly higher in ONFH patients with femoral head pre-collapse stage than collapse stage (219.3±39.7 pg/mL vs 199.6±32.5 pg/mL, P=0.007). In addition, we found that serum TNF-α levels and IL-1β levels were significantly higher in ONFH patients than healthy controls (TNF-α 1.41±0.10 pg/mL vs 1.03±0.04 pg/mL, *P*<0.001; IL-1β 3.45±0.12 pg/mL vs 1.05±0.05 pg/mL, *P*<0.001) (Table 1). However, there were no significant differences of serum C-MSF levels between the two groups (70.27±12.16 pg/mL vs 67.05±14.14 pg/mL, *P*=0.179) (Table 1).

### Correlation of serum VIP levels with radiographic progression

**Fig. 2:**
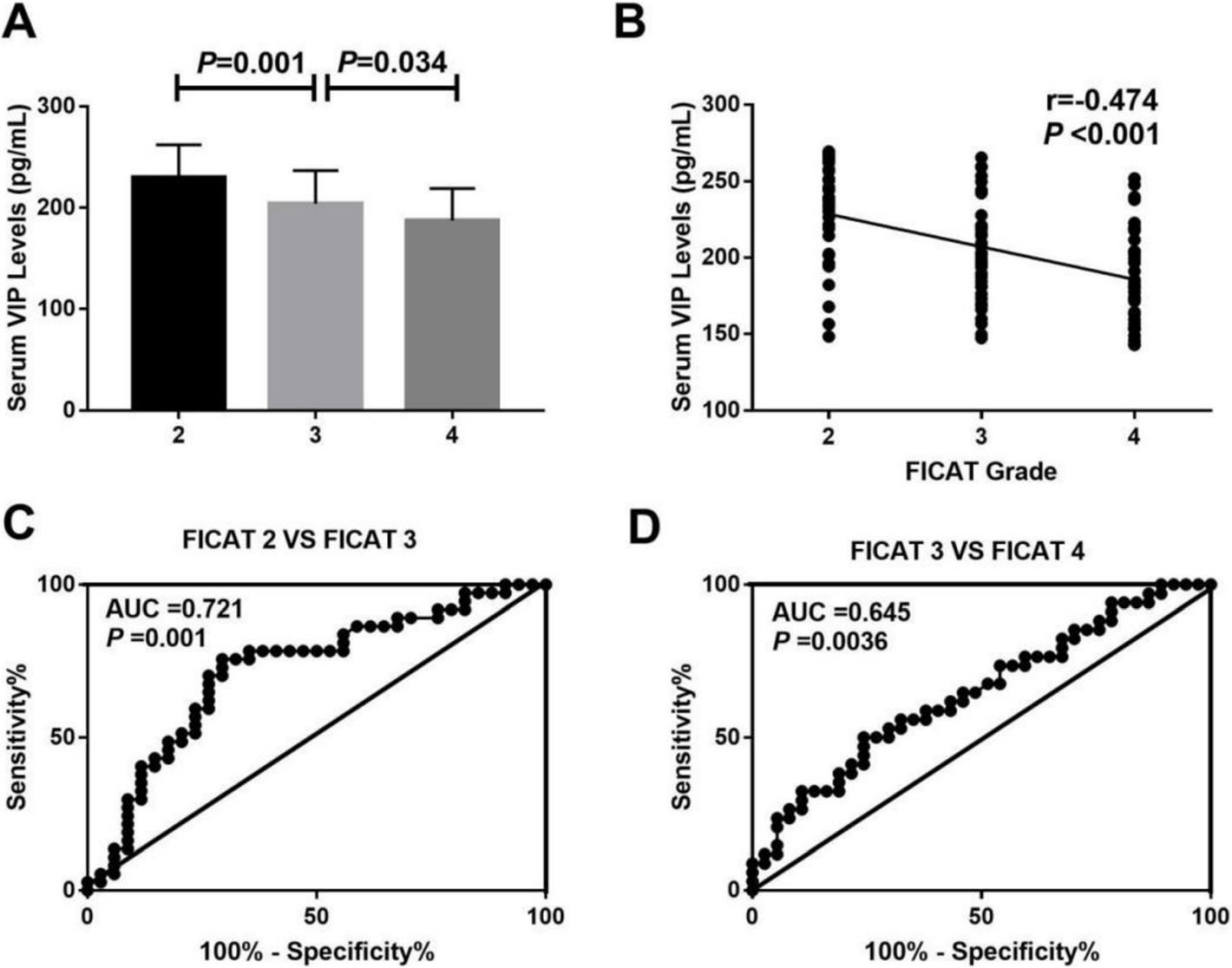
**A** Comparison of serum VIP levels among different FICAT stages. **B** Correlation of serum VIP levels with FICAT grade. **C** ROC curve analysis of VIP levels with regard to FICAT 2 vs FICAT 3. **D** ROC curve analysis of VIP levels with regard to FICAT 2 vs FICAT

The serum VIP concentrations of 105 non-traumatic ONFH patients with different FICAT grades are demonstrated in Table 2. There are 34 ONFH patients with FICAT stage 2, 37 with stage 3, and 34 with stage 4, respectively. FICAT 4 non-traumatic ONFH patients had significantly lower serum concentrations of VIP when compared with FICAT 3 (187.4±31.8 pg/mL vs 204.0±33.0 pg/mL, P=0.001) (Fig. 2A). Moreover, serum VIP levels were significantly lower in ONFH patients with FICAT 3 than FICAT 2 (204.0±33.0 pg/mL vs 230.2±32.6 pg/mL, *P*=0.034). Serum VIP levels were negatively related to FICAT stage (r=−0.474, *P*<0.001) (Fig. 2B).

**Table 2:** Association of serum VIP levels with anthropometric parameters, FICAT stage, HSS scores, and VAS scores in non-traumatic ONFH patients adjusted by age and BMI

	Serum VIP levels (pg/mL)		Serum VIP levels (pg/mL) *	
Variables	*r*	*P*	*r*	*P*
**BMI**	0.025	>0.05	/	/
**Age**	0.098	>0.05	/	/
**FICAT grade**	−0.474	<0.001	−0.415	0.001
**VAS scores**	−0.529	<0.001	−0.458	<0.001
**HSS scores**	0.473	<0.001	0.412	0.001
**TNF-α levels**	−0.455	<0.001	−0.401	0.002
**IL-1β levels**	−0.480	<0.001	−0.428	0.001

*Adjusted by age and BMI

### ROC curve analysis

We further conducted ROC curve analysis to explore the diagnostic value of VIP for radiographic progression. As shown in Fig. 2, for both FICAT grade 2 vs grade 3 and FICAT grade 3 vs grade 4, decreased VIP showed significantly AUC (AUC=0.721, *P*=0.001; AUC=0.645, *P*=0.036) (Fig. 2C, D). These findings indicate that decreased VIP may serve as a potential marker for the radiographic progression throughout all the stages of non-traumatic ONFH.

### Correlation of serum VIP levels with symptomatic severity and biochemical indices

**Fig. 3:**
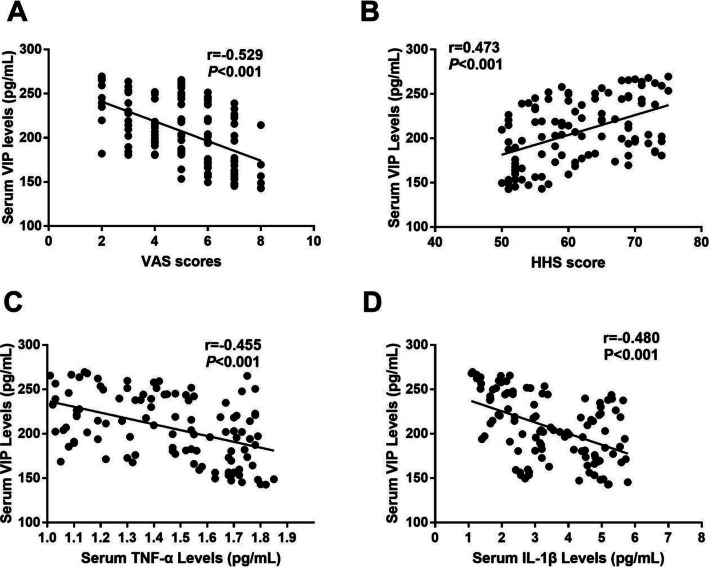
**A** Correlation of serum VIP levels with VAS scores. **B** Correlation of serum VIP levels with HHS scores. **C** Correlation of serum VIP levels with serum TNF-α levels. **D** Correlation of serum VIP levels with serum IL-1β levels

We last investigated the relationship between serum VIP levels with symptomatic severity determined by VAS and HSS, respectively. As demonstrated in Fig. 3, VIP levels in serum were negatively correlated with VAS scores (r = −0.529, *P* <0.001) (Fig. 3A) and positively associated with HSS scores (r=0.473, *P*<0.001) (Fig. 3B). We last detected the potential correlation of serum VIP levels with serum TNF-α levels and IL-1β levels; we found that serum VIP levels were both negatively correlated with serum TNF-α levels (r=−0.445, *P*<0.001) (Fig. 3C) and IL-1β levels (r=−0.480, *P*<0.001) (Fig. 3D). All the correlations remained significant after adjusting for age and BMI (Table 2).

## Discussion

This study examined the serum VIP levels associated with non-traumatic ONFH disease severity to test the potential role of VIP as biomarker for monitoring ONFH progression. We found serum VIP levels were significantly decreased in non-traumatic ONFH patients in comparison with healthy controls. Moreover, serum VIP levels were negatively associated with radiographic progression determined by FICAT classification. Further ROC curve analysis indicated that reduced VIP may serve as a potential marker for the radiographic progression throughout all the stages of non-traumatic ONFH. Last, we observed that serum VIP levels were negatively related to VAS score and positively associated with HHS score. All these findings suggested that decreased serum VIP correlated with disease progression of non-traumatic ONFH.

Treatment of hip joint preservation requires the femoral head before collapse to be successful [19]. However, ONFH is an underlying disease, and early ONFH is usually asymptomatic before collapse. MRI remains the most definitive method for the diagnosis of ONFH, as stage I ONFH cannot be detected by CT scan and radiographs. However, the high cost of MRI scans places a heavy burden on the healthcare system [20, 21], highlighting the need for convenient laboratory tests to determine ONFH progress.

VIP is a neuropeptide that is initially expressed in the intestines and lungs and then identified in neurons of the central nervous system (CNS) and endocrine and immune organs [22]. VIP has recently been identified as a potential target for the treatment of osteoarthritis (OA). Indeed, downregulation of VIP in OA synovial fluid increases the production of pro-inflammatory cytokines, and upregulation of VIP can reduce pain in OA [10]. VIP-positive nerve fibers were also detected in the periosteum, and VIP receptors were found in both osteoblasts and osteoclasts. VIP is an anabolic factor that promotes bone formation and reduces bone resorption [23].

In our study, we first found that levels of VIP in non-traumatic ONFH were lower compared with normal control, and VIP levels were also decreased in pre-collapse stage than collapse stage in ONFH patients, indicating the VIP signaling function loss may exist in ONFH. VIP is important for osteogenesis and bone repair ability, and such ability is likely impaired in ONFH patients. Further ROC analysis implicated that VIP may serve as a potential biomarker for both early and medium-late stage in ONFH patients. We last found VIP levels were negatively associated with FICAT grade and VAS score and positively related to HSS. All the correlations remained significant after adjusting for age and BMI. These findings of decreased VIP were both correlated with radiographic progression and clinical severity.

There were several limitations to be noted. First, this is a single-center study with relative small sample of Chinese population, although with strong statistical power for sample size. Further studies in different races and larger samples are needed. Second, only VIP was tested in our study; investigation of other potential peptides including NPY, PACAP, and ghrelin, and bone-related metabolic biochemical marker may reveal much more valuable information for ONFH diagnosis.

To sum up, the present study demonstrated that patients with non-traumatic ONFH have significantly lower serum levels of VIP than normal population. Decreased VIP may reflect disease severity of non-traumatic ONFH.

## Data Availability

The datasets used and/or analyzed during the current study are available from the corresponding author on reasonable request.
